# White Matter Tract Alterations in Drug-Naïve Parkinson’s Disease Patients With Impulse Control Disorders

**DOI:** 10.3389/fneur.2018.00163

**Published:** 2018-03-20

**Authors:** Mahtab Mojtahed Zadeh, Amir Ashraf-Ganjouei, Farzaneh Ghazi Sherbaf, Maryam Haghshomar, Mohammad Hadi Aarabi

**Affiliations:** ^1^Faculty of Medicine, Tehran University of Medical Sciences, Tehran, Iran; ^2^Students’ Scientific Research Center, Tehran University of Medical Sciences, Tehran, Iran

**Keywords:** impulse control disorders, Parkinson’s disease, diffusion MRI, connectometry, drug-naïve

## Abstract

Impulse control disorders (ICDs) are relatively frequent in patients with Parkinson’s disease (PD), although it is still unclear whether an underlying pathological process plays a significant role in the development of ICD in PD apart from dopaminergic replacement therapy. In this study, we have investigated alterations of white matter tract in drug-naïve PD patients with ICDs *via* diffusion MRI connectometry. Our results showed that disrupted connectivity in the complex network of dynamic connections between cerebellum, basal ganglia, cortex, and its spinal projections serves as the underlying neuropathology of ICD in PD not interfered with the contribution of dopaminergic replacement therapy. These findings provide the first evidence on involved white matter tracts in the neuropathogenesis of ICD in drug-naïve PD population, supporting the hypothesis that neural disturbances intrinsic to PD may confer an increased risk for ICDs. Future studies are needed to validate the attribution of the impaired corticocerebellar network to impulsivity in PD.

## Introduction

Impulse control disorders (ICDs) are repetitive, excessive, and compulsive behaviors, disrupting a person’s function in major areas of life (1). Prevalence of ICDs is higher among patients with Parkinson’s disease (PD) compared to normal population affecting 6–15.5% of PD patients while hitting 1.1–1.6% of the general adult population (2). Major ICDs distressing PD patients, include pathological gambling, hypersexuality, compulsive buying, and binge eating (3). In addition, other disorders have been reported in the impulsive–compulsive spectrum in PD patients, such as dopamine dysregulation syndrome, dopamine dependency syndrome, dopamine deficiency syndrome (4), punding (stereotyped, repetitive, aimless behaviors), hobbyism (e.g., artistic endeavors, excessive writing) (5), and excessive hoarding (6).

It is now well established that ICDs can be triggered by dopaminergic drugs (7). Therefore, previous studies have mainly attributed the emergence of ICDs in PD patients to the side effect of dopaminergic replacement therapy. Preliminary comparison studies have shown that ICDs are more common in PD patients on dopamine agonists than healthy controls (HC) (8–11), and untreated *de novo* PD patients manifest these behavioral phenotypes not more than general population (12, 13). However, not all PD patients on dopaminergic drugs suffer ICD. Besides other possible contributing variables, such as younger age, being unmarried, cigarette smoking, male sex, and positive family history (10, 14), it is not yet clear whether neural disturbances intrinsic to PD may confer an increased risk for ICDs. Although prevalence studies have not reached to this notion, there exist some supportive evidence. Milenkova et al. showed that PD patients without ICD perform more impulsively irrelevant of on/off treatment status (15). In addition, disinhibition failure in treated PD was revealed to be related to cortical atrophy in fronto-striatal areas (16), the key regions of the hallmark mesocorticolimbic network responsible of impulsive–compulsive behaviors (17). Similar phenotypic manifestations and neural underpinnings of ICD in PD and non-PD population are apparent in subsequent studies. Different neuroimaging studies in treated PD patients with ICD have shown various dysfunctions in the brain networks involved in decision making and risk processing, such as disconnection between anterior cingulate cortex and the striatum, increased monoaminergic activity in the medial orbitofrontal cortex, an abnormal resting-state dysfunction of the mesocorticolimbic network, etc (18–23). Consequently, it is suggested that ICD should be considered as a distinct endophenotype in PD, resulting from neuroanatomical abnormalities in impulse control regions of the brain, which would be provoked mainly by dopaminergic replacement therapy (24). However, all these studies have been conducted on PD patients already on dopaminergic treatment, so it is impossible to distinguish these findings as a reflection of treatment (25) or potential biomarkers of ICD in PD. A recent functional MRI study was designed to explore neural markers of upcoming ICD in drug-naïve early PD patients after initiation of the dopaminergic therapy. The results demonstrate that altered connectivity in salience, executive, and default-mode networks in baseline visits predict the development of ICD triggered by dopaminergic treatment (26).

In order to examine whether an underlying neuropathological process apart from medication-related effects plays a remarkable role in the establishment of ICD in PD, we investigated alterations of white matter tract in drug-naïve early PD patients with ICDs (PD-ICD) compared to PD patients without ICD (PD-nICD) and healthy controls (HC) *via* diffusion MRI connectometry.

## Materials and Methods

### Participants

Participants involved in this research were recruited from Parkinson’s Progression Markers Initiative (PPMI, http://www.ppmi-info.org/) (27). The study was approved by the institutional review board of all participating sites. Written informed consent was obtained from all participants before study enrollment. The study was performed in accordance with relevant guidelines and regulations. The participants’ PD status was confirmed by Movement Disorder Society-Unified Parkinson’s Disease Rating Scale (MDS-UPDRS) and the loss of dopaminergic neurons was observed on DAT scans. Patients were tested and confirmed negative for any neurological disorders apart from PD. Subjects were only excluded if imaging failed specific quality control criteria. 113 cases divided into three groups, (21 PD-ICD, 68 PD-nICD, and 23 HC) were recruited from baseline available diffusion imaging data from PPMI project. ICD was assessed using the Questionnaire for Impulsive–Compulsive Disorders (QUIP), which is a validated screening tool in PD patients (28). Participants in each category were matched for age, sex, and years of education. PD patients of two groups did not differ in terms of disease duration, motor severity (total UPDRS and Hoehn and Yahr stage), motor subtype (tremor versus postural instability gait difficulty), cognitive status (Montreal Cognitive Assessment), and other non-motor symptoms (REM sleep behavior disorder, excessive daytime sleepiness, and olfaction dysfunction). Although neither group showed depressive symptoms based on geriatric depression scale (29), nICD group showed significantly higher scores than ICD group. However, in the following connectometry analysis, PD-nICD did not show lower connectivity in any white matter pathways compared to PD-ICD. PD patients differed from HC in motor impairment and also only in olfactory dysfunction and depressive symptoms among all non-motor symptoms surveyed. Demographic and clinical data are represented in Table 1.

**Table 1:** Demographic information and comparison of clinical outcomes between HC and patients with PD.

Characteristic	HC (*n* = 23)	PD-ICD (*n* = 21)	PD-nICD (*n* = 68)	*P* Value	*Post hoc P* value (PD groups)
Age, mean (SD) [95% CI], years	58.3 (10.5) [53.7–62.8]	57.7 (9.8) [53.3–62.2]	59.1 (9.5) [56.9–61.5]	0.801^a^	0.829
Female/male, No. (% male)	11/12 (52.2)	7/14 (66.6)	24/44 (64.7)	0.511^b^	0.869
Left-handed/right-handed, No. (% right-handed)^x^	3/19 (82.6)	1/19 (90.5)	7/58 (85.3)	0.925^b^	0.741
Education, mean (SD) [95% CI], years	14.6 (2.8) [13.4–15.8]	14.6 (2.7) [13.4–15.8]	15.4 (2.9) [14.6–16.0]	0.374^c^	0.307
Disease duration, mean (SD) [95% CI], years	…	10.4 (10.5) [5.6–15.1]	5.8 (5.3) [4.6–7.1]	0.272^d^	…
Hoehn and Yahr stage, mean (SD)	…	1.6 (0.5)	1.6 (0.5)	0.891^b^	…
Tremor score, mean (SD)	0.063 (0.84)	0.446 (0.300)	0.458 (0.282)	<0.001^c^^,^^e^	0.631
PIGD score, mean (SD)	0.052 (0.108)	0.229 (0.192)	0232 (0.164)	<0.001^c^^,^^e^	0.702
MDS-UPDRS part I score, mean (SD)	2.8 (1.6)	6.2 (3.1)	4.4 (3.2)	<0.001^c^^,^^e^	0.007
MDS-UPDRS part II score, mean (SD)	2.2 (2.7)	5.1 (4.5)	5.2 (5.0)	0.008^c^^,^^e^	0.880
MDS-UPDRS part III score, mean (SD)	0.6 (1.2)	21.1 (8.5)	21.4 (8.8)	<0.001^c^^,^^e^	0.973
MDS-UPDRS total score, mean (SD)	5.6 (3.6)	32.4 (10.7)	31.0 (11.2)	<0.001^c^^,^^e^	0.492
MoCA score, mean (SD)	28.4 (1.1)	27.2 (2.0)	27.6 (2.0)	0.196^c^	0.450
GDS score, mean (SD)	4.7 (1.1)	3.8 (1.4)	4.6 (1.2)	0.046^c^	0.033
UPSIT score, mean (SD)	33.5 (4.6)	22.1 (8.1)	23.2 (8.4)	<0.001^c^^,^^e^	0.474
RBD score, mean (SD)	3.2 (2.3)	4.7 (3.2)	3.8 (2.4)	0.357	0.342
ESS score, mean (SD)	6.2 (4.3)	6.8 (3.3)	6.1 (3.3)	0.739	0.478

**Type of ICD**					
Hypersexuality		1 (4.5%)			
Compulsive buying		1 (4.5%)			
Compulsive eating		8 (36%)			
Hobbies		2 (9%)			
Punding		5 (23.5%)			
Walking or Driving + hobbies		2 (9%)			
Compulsive eating + punding		1 (4.5%)			
Compulsive buying + hobbies		1 (4.5%)			
Compulsive buying + eating + punding		1 (4.5%)			

*HC, healthy controls; PD, Parkinson disease; PIGD, postural instability and gait difficulty; MDS-UPDRS, movement disorder society-sponsored revision of the unified Parkinson’s disease rating scale; MoCA, montreal cognitive assessment; GDS, geriatric depression scale; UPSIT, University of Pennsylvania smell identification test; ESS, epworth sleepiness scale; RBD, REM sleep behavior disorder*.

*^a^Based on one-way ANOVA*.

*^b^Based on χ^2^ test*.

*^c^Based on Kruskal–Wallis test*.

*^d^Based on Mann–Whitney U test*.

*^e^Post hoc analysis showed significant differences between HC and two PD groups*.

*^x^Others were mixed-handed*.

### Data Acquisition

Data used in the preparation of this article were obtained from the PPMI database (www.ppmi-info.org/data) (27). This dataset was acquired on a 3 T Siemens scanner, producing 64 diffusion MRI (repetition time = 7,748 MS, echo time = 86 ms; voxel size: 2.0 mm × 2.0 mm × 2.0 mm; field of view = 224 mm × 224 mm) at *b* = 1,000 s/mm^2^ and one b0 image along with a 3D T1-weighted structural scan (repetition time = 8.2 ms, echo time = 3.7 ms; flip angle = 8°, voxel size: 1.0 mm × 1.0 mm × 1.0 mm; field of view = 240 mm, acquisition matrix = 240 × 240).

### Diffusion MRI Processing

The diffusion MRI data were corrected for subject motion and eddy current distortions using Explore DTI toolbox, which reorients the B-matrix in the stage of realigning the images to preserve the orientational information correctly (30). Orienting B-matrix is a simple, but indeed essential step in avoiding bias in diffusion measures especially in PD patients who are susceptible to move during the scans (31). We also performed quality control analysis on the subject’s signals based on the goodness-of-fit value given in q-space diffeomorphic reconstruction (QSDR) of fibers (32). Each QSDR reconstruction file has a goodness-of-fit value quantified by R2. For example, an R82 indicates a goodness-of-fit between of the subject and template of total 0.82. We excluded cases in which the R2 value did not reach a threshold of 0.6 otherwise.

### Between Groups Analysis

The diffusion data were reconstructed in the MNI space using QSDR to obtain the spin distribution function (SDF), to detect the differences between groups (PD-ICD, PD-nICD, and HC).

Connectometry (33) is a novel approach in the analysis of diffusion MRI signals that simply tracks the difference of white matter tracts between groups, or correlation of white matter fibers with a variable of interest. Connectometry approach extracts the SDF in a given fiber orientation, as a measure of water density along that direction. There is a multitude of diffusion indices derived from spin density, i.e., SDF, quantitative anisotropy (QA) being one of them. QA of each fiber orientation gives the peak value of water density in that direction. We used diffusion MRI connectometry to identify white matter tracts in which QA was significantly different between three groups. Resulting uncorrected output was corrected for multiple comparisons by false discovery rate (FDR). A deterministic fiber tracking algorithm (34) was conducted along the core pathway of the fiber bundle to connect the selected local connectomes. Tracts with QA > 0.1, angle threshold lesser than 40^o^, and tract length greater than 40 mm were included. To estimate the FDR, a total of 2,000 randomized permutation was applied to the group label to obtain the null distribution of the track length. Permutation testing allows for estimating and correcting the FDR of type-I error inflation due to multiple comparisons. The analysis was conducted using publicly available software DSI Studio (http://dsi-studio.labsolver.org).

### Statistical Analysis

Demographic and clinical data were analysed using SPSS version 22 (IBM Corp., Armonk, NY, USA). *P* values less than 0.05 were considered to be statistically significant. Pearson’s chi-square was used to assess nominal variables across groups. Mann–Whitney *U* test was used to assess differences between two groups, and Kruskal–Wallis test or one-way ANOVA was used for multiple comparisons for three groups.

## Results

### PD-ICD Patients Versus PD-nICD Patients

**Figure 1:**
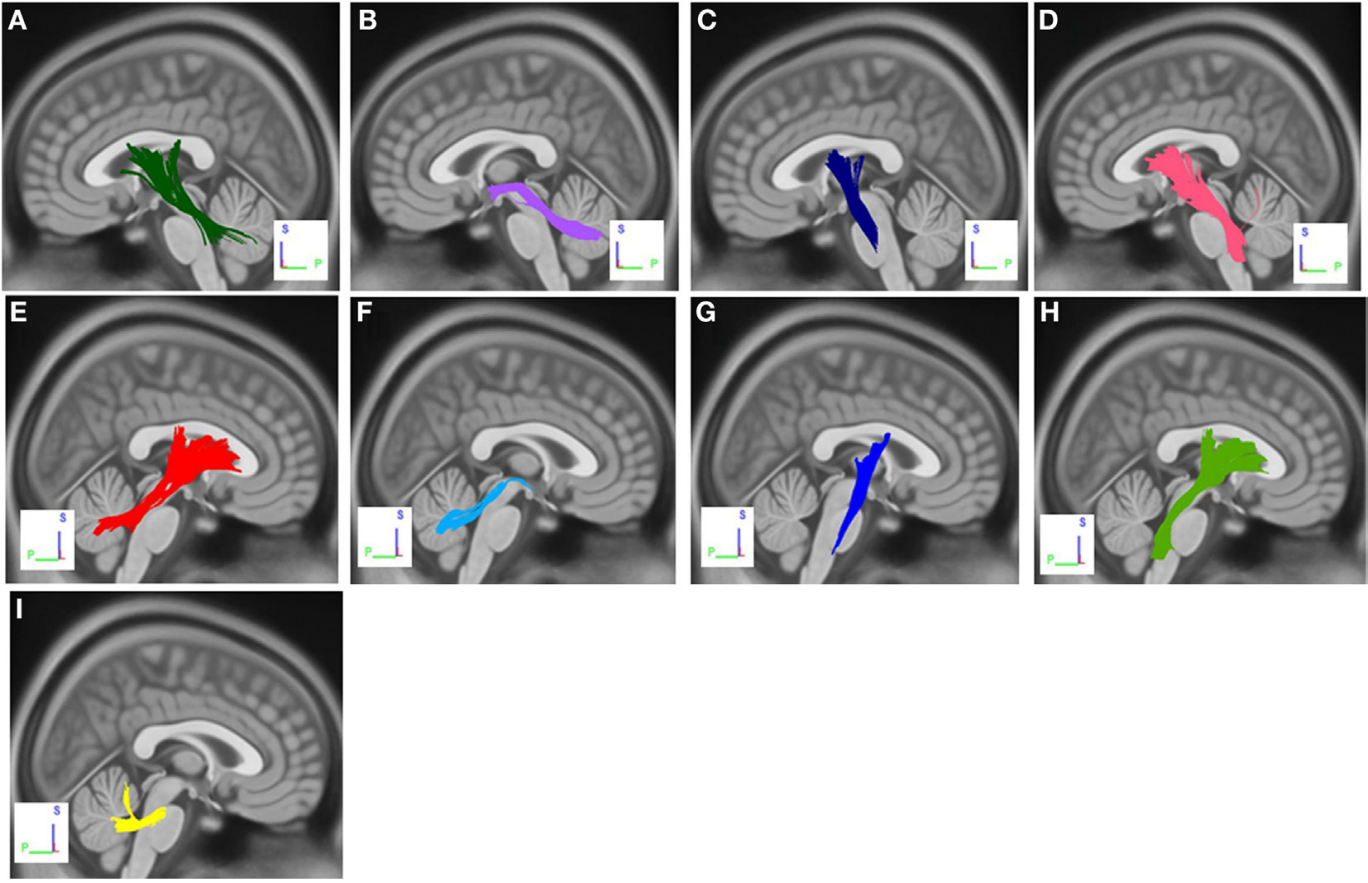
White matter pathways with significantly reduced anisotropy in PD-ICD patients compared to Parkinson’s disease-nICD [false discovery rate = 0.008]. **(A)** Left cortico-thalamic tract, **(B)** left superior cerebellar peduncle (SCP), **(C)** left corticospinal tract (CST), **(D)** left cortico-pontine tract, **(E)** right cortico-thalamic tract, **(F)** right SCP, **(G)** right CST, **(H)** right cortico-pontine tract, and **(I)** middle cerebellar peduncle. The results are overlaid on ICBM152 (mni_icbm152_t1) from the McConnell Brain Imaging Centre using DSI-STUDIO software.

The group differences between PD-ICD patients and PD-nICD are shown in Figure 1. Compared with PD-nICD patients, PD-ICD patients showed decreased connectivity in the left and right cortico-thalamic tract, the left and right cortico-pontine tract, the left and right corticospinal tract (CST), the left and right superior cerebellar peduncle (SCP), and the left and right middle cerebellar peduncle (MCP) (FDR = 0.008).

### PD-nICD Versus HC

**Figure 2:**
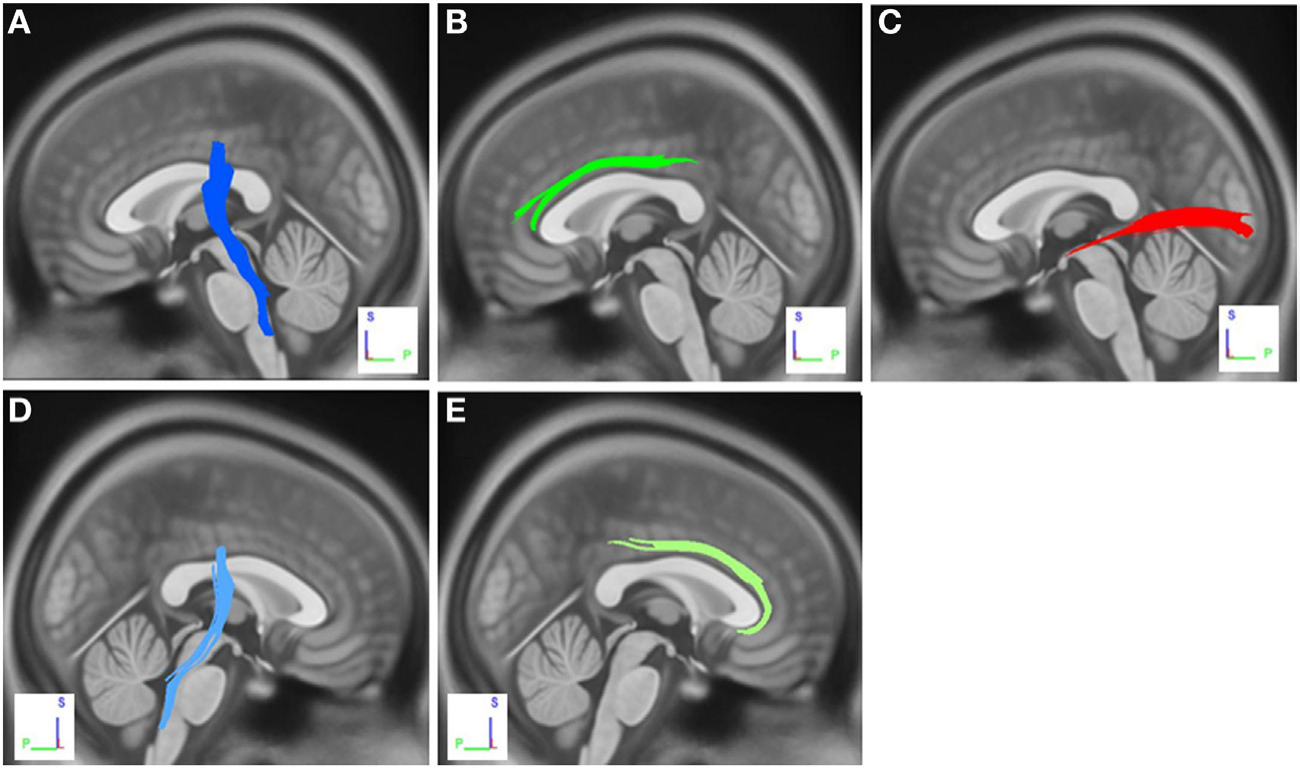
White matter pathways with significantly reduced anisotropy in Parkinson’s disease-nICD compared to healthy controls (false discovery rate = 0.001). **(A)** Left corticospinal tract, **(B)** left cingulum, **(C)** left inferior longitudinal fasciculus, **(D)** right corticospinal tract, and **(E)** right cingulum. The results are overlaid on ICBM152 (mni_icbm152_t1) from the McConnell Brain Imaging Centre using DSI-STUDIO software.

The group differences between PD-nICD patients and HC are shown in Figure 2. The differences were that connectivity in HC was higher than that in PD-nICD in the left inferior longitudinal fasciculus (ILF), the left and right CST, and the left and right cingulum (FDR = 0.001).

### PD-ICD Patients Versus HC

The differences were that connectivity in HC was higher than that in PD-ICD patients in the left and right ILF, genu and body of the corpus callosum (CC), the left and right CST, the left SCP, and the left and right cingulum (FDR = 0.002).

## Discussion

This study revealed that compared to HC, drug-naïve PD patients have microstructural changes in the CST, ILF, and cingulum. PD-ICD patients also showed additional pathways, i.e., genu and body of CC and SCP compared to HC. These tracts are commonly presented in the literature in relation to various motor and non-motor symptoms of PD, such as olfaction dysfunction, mood and sleep dysregulations, and cognitive decline [reviewed in Hall et al. (35)].

Neural contributions of impulsivity in PD have recently grabbed attention, and some studies have investigated white tract alterations in PD patients with ICDs. In a DTI study, Canu et al. compared white matter microstructure of PD patients with and without punding, at the time when they were on dopaminergic medication. They showed that punding in PD patients is associated with the disconnection between midbrain, limbic, and white matter tracts projecting to the frontal cortex (36). Yoo et al. also indicated some structural alterations in PD-ICD patients, especially in the CC (22). Another study using DTI and resting-state fMRI showed that PD-ICD patients had more severe involvement of frontal, mesolimbic, and motor circuits (23). These results suggest that ICD might be the result of a disconnection between sensorimotor, associative, and cognitive networks in PD patients (23).

fMRI studies showed that ventral striatum and anterior cingulate might be associated with risk and reward-related behaviors and decision making (37, 38). In a risk-taking task, PD-ICD patients showed decreased anterior cingulate and orbitofrontal cortex activity in comparison to PD-nICD. Moreover, pharmacological manipulation (using dopamine agonists) resulted in decreased ventral striatal activity in PD-ICD group, compared with PD-nICD group (38). An experiment with gambling-related visual cue showed that in PD patients with pathological gambling, there is altered activity in the ventral striatum, anterior cingulate cortex, and frontal gyri (39). Resting-state fMRI studies also indicated a functional disconnection between a striatal associative area (the left putamen) and cortical associative (inferior temporal) and limbic regions (anterior cingulate) in PD patients with ICD compared to PD-nICD group (40).

Regarding gray matter (GM), studies are not consistent. Some studies showed that PD patients with ICD had a reduction in cortical thickness of fronto-striatal regions when compared to other PD patients (41). Moreover, Biundo et al. indicated that the level of GM alterations is associated with the severity of ICDs in PD patients (42). Interestingly, Tessitore et al. had completely different results. They indicated that PD-ICD patients have thicker orbitofrontal and anterior cingulate cortices, in comparison to PD-nICD. They also showed that these abnormalities were positively correlated with ICD severity (26). Finally, another study showed relatively preserved GM in PD patients with ICD when compared to PD patients without such disorder (43).

Most studies have compared brain alterations of PD patients with and without ICD at the time they were on dopaminergic medication (44). However, evidence from our study on white matter microstructural alterations in drug-naïve PD patients supports the hypothesis that these abnormalities may be due to neurodegenerative processes intrinsic to PD. These changes might be an independent risk factor for developing ICDs in PD patients and may interact with chronic treatment with dopamine agonists. Other studies have shown that decreased dopamine transporter availability might predict the risk of future ICD behaviors in drug-naïve PD patients who are going to take dopamine replacement therapy in the future (45). Variend et al. also showed that lower level of dopamine transporters in striatal regions might predate the incidence of ICDs in PD patients after the beginning of dopaminergic treatment and may be an independent risk factor for punding behaviors (46). These results highlight the fact that PD itself may play a significant role in developing ICDs in parkinsonian patients.

Cerebellum participates in higher order functions of cognition and emotion by means of bidirectional communications to limbic and paralimbic regions and neocortex, especially prefrontal and posterior parietal areas (47–50). Several behavioral disorders such as impulsive actions are reported following cerebro-cerebellar circuitry damage (51, 52). Disruption of the parieto-ponto-cerebellar loop through lower connectivity in MCP and cerebello-basal ganglia-thalamo-cortical loop *via* lower connectivity in SCP was demonstrated in relation to ICD in our cohort of PD patients. These loops process information in cognitive, emotional, and behavioral domains. In this complicated network, cerebellum, cortex, and basal ganglia have integrated roles in reinforcement learning anchored to reward predictions of dopamine signals in the striatum (53). The interplay between these structures underlies the complex motor and cognitive functions. Evidence regarding disruption of this system is multitude with respect to motor and cognitive features of PD (53). In particular, the cerebellum is strongly connected to the striatum *via* output projections of SCP to the thalamus (54). Since striatum as a part of mesocorticolimbic network plays the central role in the pathology of misbehaviors such as addiction and impulsion–compulsion linked to reward learning (17), it seems that cerebellar corroboration in this scenario is often neglected. Although the vast network of cerebro-cerebellar communications is often assumed to be confined to multi-synaptic pathways by means of pontine and thalamic nuclei, simultaneous activation of corticospinal fibers plays a definitive role in relaying feedbacks to the learning processes (53). The contribution of the multi-synaptic cortico-cerebellar network as underlying neuropathology of ICD in early PD without the interference of dopaminergic drugs is a novel and promising result that should be more addressed in future studies.

Some methodological limitations should be considered when interpreting our results, such as small sample size of participants, no-follow up assessments, and not to take into account other risk factors attributed to ICD such as previous histories of addiction and family histories of ICD. Although PD-ICD and PD-nICD patients did not differ in terms of motor and non-motor symptoms, PD patients showed worse scores in screening tests of olfaction function and depressive symptoms compared to healthy controls. This may account for observed alterations in neural connectivity comparing PD-ICD with HC. Future studies are needed to validate if the presented white matter tracts by this preliminary study serve as possible neural markers of ICD in PD. Measurement of the correlation of severity of ICD symptoms with MRI parameters will add valuable information.

In conclusion, this is the first study that investigates the alteration of white matter tracts relative to impulsive–compulsive behaviors in drug-naïve PD patients. Our results showed that disrupted connectivity in the complex network of dynamic connections between cerebellum, basal ganglia, cortex, and its spinal projections serves as the underlying neuropathology of ICD in PD not interfered with the contribution of dopaminergic replacement therapy. Association of these novel pathways provides a potential explanation of why dopamine agonists can lead to an unconscious bias toward risk in some individuals suffering PD. Further studies can evaluate this hypothesis and bring about more evidence, to diagnose ICDs in early stages of PD.

## Ethics Statement

All procedures performed here, including human participants were in accordance with the ethical standards of the institutional research committee and with the 1964 Helsinki declaration and its later amendments or comparable ethical standards. *Informed consent*: informed consent was obtained from all individual participants included in the study.

## Author Contributions

MZ, AA-G, FS and MA contributed to the conception and design of the study. MZ, MH, FS, and MA contributed to data collection and analysis. MZ, AA-G, FS, and MA contributed to writing the manuscript.

## Conflict of Interest Statement

The authors declare that the research was conducted in the absence of any commercial or financial relationships that could be construed as a potential conflict of interest.
